# Radiation Grafting of a Polymeric Prodrug onto Silicone Rubber for Potential Medical/Surgical Procedures

**DOI:** 10.3390/polym12061297

**Published:** 2020-06-05

**Authors:** Hector Magaña, Claudia D. Becerra, Aracely Serrano-Medina, Kenia Palomino, Giovanni Palomino-Vizcaíno, Amelia Olivas-Sarabia, Emilio Bucio, José M. Cornejo-Bravo

**Affiliations:** 1 Facultad de Ciencias Químicas e Ingeniería, Universidad Autónoma de Baja California, Calzada Universidad 14418, Parque Industrial Internacional Tijuana, Tijuana 22390, Mexico; hector.magana@uabc.edu.mx (H.M.); cbecerra30@uabc.edu.mx (C.D.B.); kenia.palomino@uabc.edu.mx (K.P.); 2 Facultad de Medicina y Psicología, Universidad Autónoma de Baja California, Tijuana 22390, Mexico; aracely.serrano@uabc.edu.mx; 3 Facultad de Ciencias de la Salud, Universidad Autónoma de Baja California, Boulevard Universitario 1000, Valle de las Palmas, Tijuana 22260, Mexico; gpalomino@uabc.edu.mx; 4 Centro de Nanociencias y Nanotecnología, Universidad Nacional Autónoma de México, Apdo. Postal 14, Ensenada 22800, Mexico; aolivas@cnyn.unam.mx; 5 Departamento de Química de Radiaciones y Radioquímica, Instituto de Ciencias Nucleares, Universidad Nacional Autónoma de México, Circuito Exterior, Ciudad Universitaria, CDMX 04510, Mexico

**Keywords:** grafting, polymeric prodrug, medical devices, medical/surgical procedures, pH-dependent release, drug delivery

## Abstract

Silicone rubber (SR) is a material used for medical procedures, with a common example of its application being in implants for cosmetic or plastic surgeries. It is also an essential component for the development of medical devices. SR was functionalized with the polymeric prodrug of poly(2-methacryloyloxy-benzoic acid) (poly(2MBA)) to render the analgesic anti-inflammatory drug salicylic acid by hydrolysis. The system was designed by functionalizing SR films (0.5 cm × 1 cm) with a direct grafting method, using gamma irradiation (^60^Co source) to induce the polymerization process. The absorbed dose (from 20 to 100 kGy) and the monomer concentration (between 0.4 and 1.5 M) were critical in controlling the surface and the bulk modifications of SR. Grafting poly(2MBA) onto SR (SR-*g*-2MBA) were characterized by attenuated total reflectance Fourier transform infrared spectroscopy, thermogravimetric analysis, scanning electron microscopy/energy-dispersive X-ray spectrometry, fluorescence microscopy, the contact angle, and the swelling. SR-g-2MBA demonstrated the drug’s sustained and pH-dependent release in simulated physiological mediums (pH = 5.5 and 7.4). The drug’s release was quantified by high-performance liquid chromatography and confirmed by gas chromatography–mass spectrometry. Finally, cytocompatibility was demonstrated in murine fibroblast and human cervical cancer cell lines. The developed systems provide new polymeric drug release systems for medical silicone applications.

## 1. Introduction

Medical devices are essential to many surgical procedures [1]; therefore, it is important to have implantable devices that help reduce the inflammatory processes caused by tissue lesions or immunological responses, which have the consequence of prolonging a patient’s hospital stay [2,3].

Another negative effect related to these devices is bacterial or fungal infections either caused by poor sterilization of the device or acquired in hospitals [4,5,6]. Innovative solutions of the last decade include the development of combination products, which are defined as a combination between a medical device and a drug and which allow for the targeted release of drugs at the surgical site [7,8]. The materials used for medical devices include silicone, polypropylene, poly(vinyl chloride), and polyurethanes [9]. 

Silicone is a material that is widely used in aesthetic surgeries, such as those on the buttocks or breasts. Therefore, controlled drug-release systems (anti-inflammatory, analgesic, or antimicrobial), incorporated or loaded in the silicone, are an attractive strategy for solving the problems associated with these types of medical procedures [10,11]. 

Gamma radiation (specifically ^60^Co) is a technique for medical device modification, and it is used to graft or functionalize polymers on the surface of a device and to achieve drug and enzyme loading/release [12,13]. The method is based on the irradiation of the materials with gamma rays, which generates radicals on the surface that initiate the grafting polymerization process (the direct method) [14]. The advantages of this method include avoiding the use of a polymeric chemical initiator and the ability to obtain a sterilized product [15]. This technique has been used with catheters [16], medical gauze [17], and suture-thread modifications [18]. 

Some polymers that have been grafted onto devices include poly(acrylic acid), poly(vinyl caprolactam), and poly(2-methacryloyloxy-benzoic acid) (poly(2MBA)). The latter is a polymer prodrug that, with the hydrolysis of a labile ester bond on the pendant groups [19], releases salicylic acid for an extended period. Salicylic acid is a drug already known for its activities as an analgesic, anti-inflammatory, antithrombotic, and antimicrobial agent [20,21]. This type of polymeric prodrug (of the salicylate family) has been studied in other systems, such as catheters [22], sutures [23], hydrogels [24], tablets [25], and films [26]. 

These systems expressed antimicrobial activities against certain strains, including *Staphylococcus epidermidis, Pseudomonas aeruginosa,* and *Staphylococcus aureus*. The previous results supported the use of polymeric prodrugs of salicylic acid in devices to prevent bacterial colonization and to prevent biofilms from forming on the materials [27]. 

The objective of this work was to prepare and evaluate a combined product (a medical device–drug), employing the covalent incorporation of poly(2MBA) in silicone that is used for surgical procedures through graft polymerization by gamma radiation. We determined to what extent the radiation dose and monomer concentration (2MBA) were critical in the grafting degree of the functionalization on silicone. The grafted materials (SR-*g*-2MBA) were characterized by attenuated total reflectance Fourier transform infrared spectroscopy (FTIR-ATR), microscopy (scanning electron microscope/energy-dispersive X-ray spectrometry (SEM-EDX) and fluorescence), thermogravimetric analysis (TGA), and the contact angle. 

The kinetics of the salicylic acid release were evaluated at different pH values, quantified by high-performance liquid chromatography (HPLC), and confirmed by gas chromatography–mass spectrometry (GC-MS). Finally, we evaluated the toxicity of the materials in a cell culture.

## 2. Materials and Methods 

### 2.1. Materials 

Silicone rubber (SR) (1 mm in thickness) was provided by Goodfellow (HuntingdonUK). We washed the SR with ethyl alcohol for 24 h to remove the impurities and dried it under a vacuum (60 °C) for 3 h. All the synthesis reagents (methacrylic anhydride, salicylic acid, dimethylaminopyridine (DMAP), dichloromethane, triethylamine, magnesium sulfate, ethyl ether, p-dioxane, ethanol, toluene, and petroleum ether) were from Sigma-Aldrich (San Luis, MO, USA), DMEM medium, fetal bovine serum, streptomycin solution, gentamicin, HPLC acetonitrile and HPLC methanol, and distilled water were used as received Sigma-Aldrich (San Luis, MO, USA). The MTT kit was from Roche (Basel, Switzerland)

### 2.2. Synthesis of 2MBA

The 2MBA synthesis was carried out using a previously reported method [28]. Briefly, methacrylic anhydride (7.50 g) was added drop-wise to a solution of salicylic acid (6.91 g) and DMAP (0.49 g) in 20 mL of dichloromethane and 8.42 mL of triethylamine at 0 °C. The organic phase was extracted with ethyl ether, dried with magnesium sulfate, filtered, and finally concentrated in vacuo. The residue was purified with p-dioxane and petroleum ether, and the final product was a white solid that was characterized by FTIR-ATR and RMN-^1^H. 

### 2.3. Grafting Process (SR-g-2MBA)

SR films (0.5 cm × 1 cm) were put in contact with the monomer solution (toluene) in glass ampules. The samples were irradiated with a ^60^Co source in the dose interval from 20 to 100 kGy, and the monomer concentration ranged from 0.4 to 1.5 M. The films were washed in ethanol to remove the unrealized graft of poly(2MBA) or homopolymers. The modified SR films were dried under a vacuum at 60 °C. Finally, the graft percentage of the films was calculated was using the following gravimetry equation (Equation (1)) [12]:

(1) $$Grafting\;\left(\%\right)=\left(\frac{W-W_{o}}{W_{o}}\right)\left(100\right)$$

where *Wo* and *W* represent the mass of the SR film before and after grafting, respectively.

### 2.4. Characterization of SR-g-2MBA Films

#### 2.4.1. TGA and FTIR-ATR Analysis

Thermogravimetric analysis was evaluated with a TGA Q50, TA Instruments (New Castle, DE, USA), under an inert atmosphere, with a temperature range of 25–800 °C and a heating rate of 10 °C/min. The SR and modified films (12.1%, 18.7%, and 31.1% grafts) were characterized by FTIR-ATR (4000–650 cm^−1^) using a PerkinElmer Spectrum 100 spectrometer, PerkinElmer Cetus Instruments (Norwalk, CT, USA) with 16 scans.

#### 2.4.2. SEM-EDX and Fluorescence Analysis

Cross-sections and surfaces of SR and SR-*g*-2MBA films (0.5 cm × 1 cm) were analyzed using a scanning electron microscope, JSM-5300, JEOL (Akishima, TYO, Japan) coupled with energy dispersive X-Ray spectrometry (EDX). Fluorescence microscopy was conducted for the films, using a Nikon Ti Eclipse microscope (Minato, TYO, Japan) and applying a 4′,6-diamidino-2-phenylindole (DAPI) filter (10×). 

#### 2.4.3. Contact Angle and Swelling Test

The contact angle test was performed using a Kruss DSA 100 device (Matthews, NC, USA), in which unmodified and modified films were used. Then, water was dropped on the surface of the films in five different areas of the material to determine the contact angle. Regarding the swelling test, films with and without grafting were placed in 20 mL of distilled water at room temperature and weighed at different time intervals (15 to 240 min). The following equation, Equation (2) [29], determined the swelling percentage:

(2) $$Swelling=\left(\frac{W_{s}-W_{d}}{W_{d}}\right)\left(100\right)$$

where $W_{s}$ corresponds to the weight of the swollen film and $W_{d}$ corresponds to the weight of the dry film.

### 2.5. Salicylic Acid Release and Characterization (HPLC and GC-MS)

SR and SR-*g*-2MBA (10–20 mg; 0.5 cm × 1 cm) were exposed at 5 mL of phosphate-buffered saline (PBS) (pH = 5.5 and 7.4) and incubated at 37 °C in a Mini Shaker VWR (West Chester, PA, USA) at constant agitation (100 rpm). Sampling was performed at predetermined times. We analyzed the salicylic acid released with an HPLC Ultimate 3000 Thermo Scientific (Waltham, MA, USA) spectrophotometer using UV detection (296 nm). A Thermo Scientific Hypersil Gold column (250 mm × 4.6 mm at 45 °C) (Waltham, MA, USA) was used with a mobile phase of acetonitrile and 30:70 PBS buffer, with an isocratic flow of 1.2 mL/min and an injection volume of 10 µL. The salicylic acid concentration was determined by a calibration curve. 

The proportion of the grafted drug was determined by Equation (3) [26]:

(3) $$Salicylic\;acid\;\left(mg/g\right)=\left(\frac{Grafting\;\left(\%\right)}{\left(100+Grafting\left(\%\right)\right)\left(\;MW_{2MBA)}\right)}\right)\left(MW_{Salicylic\;acid}\right)\left(1000\right)$$

Release profiles were analyzed by zero- and first-order kinetics models using Excel (Microsoft Office). 

The presence of the salicylic acid in the release media was confirmed by GC-MS with a gas chromatograph, model TRACE 1310 Thermo Scientific (Waltham, MA, USA), coupled to a single quadrupole mass spectrometer, model ISQ LT Thermo Scientific (Waltham, MA, USA). A portion of the release medium was extracted with ethyl ether, resuspended, dissolved in methanol, and injected into the chromatograph. The temperature of the transfer line was 250 °C, and the ion source temperature was 240 °C. The oven temperature was 120 °C. We started the column heating ramp, maintaining this temperature for 1 min. Then, we raised the temperature to 280 °C at a speed of 40 °C/min and maintained this temperature for 10 min. Helium was used as the carrier gas, with a flow rate of 1 mL/min.

### 2.6. Cytocompatibility Test

We conducted the cytocompatibility test with human cervical cancer cell line HeLa (ATCC CCL-2) and murine embryonic fibroblast cell line BALB/3T3 (ATCC CCL-163). The tests were performed in 96-well half-area plates with 30,000 cells mL^−1^ in DMEM medium complement with fetal bovine serum medium (10%), streptomycin solution (1%), and 10 µg/mL of gentamicin. After 12 h of incubation, SR and SR-*g*-2MBA films (0.2 × 0.25 cm^2^) were put in contact with the cells and maintained in a humidified atmosphere at 5% CO_2_ and 37 °C. The films were removed 24 h later and an MTT Kit (Roche, Switzerland) was used to quantify the cell proliferation. A negative control was used with cells without films, and all experiments were performed in triplicate. Finally, the absorbances were determined with a microplate reader at a wavelength of 620 nm Multiskan FC, Thermo Scientific, (Waltham, MA, USA). The cytocompatibility of the films was determined by Equation (4) [26]:

(4) $$Cytocompatibility\;\left(\%\right)=\left(\frac{Abs_{Sample}}{Abs_{Control}}\right)\left(100\right)$$

Statistical analysis (one-way ANOVA) was performed with GraphPad Prism (San Diego, CA, USA).

## 3. Results and Discussion

### 3.1. Synthesis of SR-g-2MBA 

**Figure 1:**
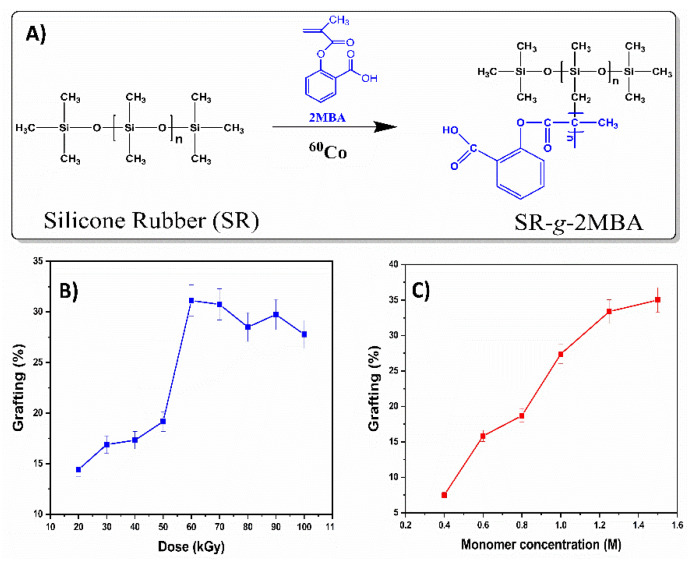
(**A**) Schematic representation of 2MBA grafting onto silicone rubber (SR); dependence of grafting percentage on (**B**) absorbed dose and (**C**) monomer concentration.

The graft polymerization by gamma irradiation successfully achieved the functionalization of 2MBA on SR (Figure 1A). The experiments showed an increase in the graft percentages at greater irradiation doses, and the maximum graft was at 60 kGy (Figure 1B). The higher gamma irradiation increased the breaking of the covalent bonds in the structure of the silicone films. In consequence, there was a higher radical formation to graft 2MBA [30]. The increasing monomer concentrations experiment demonstrated a growth in the graft percentage in the intervals between 0.4 and 1.5 M (Figure 1C). A higher graft percentage was obtained at 1.5 M (35%). The films with graft percentages greater than 20% showed a more whitish appearance and greater rigidity.

### 3.2. Characterization of SR-g-2MBA

#### 3.2.1. TGA and FTIR-ATR

**Figure 2:**
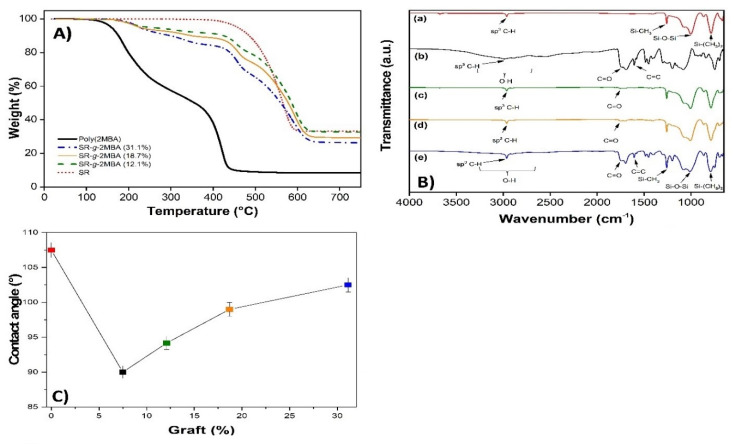
(**A**) Thermogravimetric analysis (TGA) scans of SR; SR-*g*-2MBA with 12.1%, 18.7%, and 31.1% graft; and poly(2MBA). (**B**) FTIR-ATR spectra of SR (a), poly(2MBA) (b), SR-*g*-2MBA 12.1% (c), SR-*g*-2MBA 18.1% (d), and SR-g-2MBA 31.1% (e). (**C**) Water contact of SR and SR-*g*-2MBA with grafting from 7.1% to 31.1%.

We evaluated the thermal stability of SR, poly(2MBA), and (SR-*g*-2MBA) (Figure 2A). SR presented a thermal degradation at 565 °C [31], and poly(2MBA) presented two degradations: the first at 220 °C and the second at 395 °C [28]. SR-*g*-2MBA with three different graft percentages (12.1%, 18.7%, and 31.1%) showed three thermal degradations. The first and second degradation corresponded to the poly(2MBA) and a higher loss at higher grafting, while the last corresponded to SR. Functionalization of the silicone was also confirmed by infrared spectroscopy (Figure 2B). SR presented three principal bands [32]: C–H, Si–CH_3_, and Si–O at 2978, 1263, and 1000 cm^−1^, respectively (Figure 2B(a)). The modified films with 12.1%, 18.7%, and 31.1% grafts showed the same signals as SR but also a band at 1735 cm^−1^, which is characteristic of a carbonyl group (C=O). This signal belongs to the ester and carboxylic acid group of poly(2MBA) grafted onto SR (Figure 2B(c–e)). At a greater graft (31.1%), the intensity in the poly(2MBA) bands increased (Figure 2B (b)) and even the double bond of the aromatic region was more apparent. This demonstrates the silicone modification by FTIR-ATR and TGA.

#### 3.2.2. Contact Angle

In Figure 2C, we observe that SR has a contact angle of 107°, corresponding to a hydrophobic surface [33]. The films modified with 7.1% present a contact angle of 91°. This is due to the poly(2MBA) graft that contains polar groups (carboxylic acid and ester), favoring interactions with water [34]. However, films with a graft from 12.1% to 31.1% showed an increase in the contact angle (up to 104°) due to a large amount of polymer grafted onto the surface. Consequently, the intramolecular interactions were favored and the interactions with the aqueous medium decreased [35]. 

#### 3.2.3. SEM-EDX and Fluorescence Analysis

**Figure 3:**
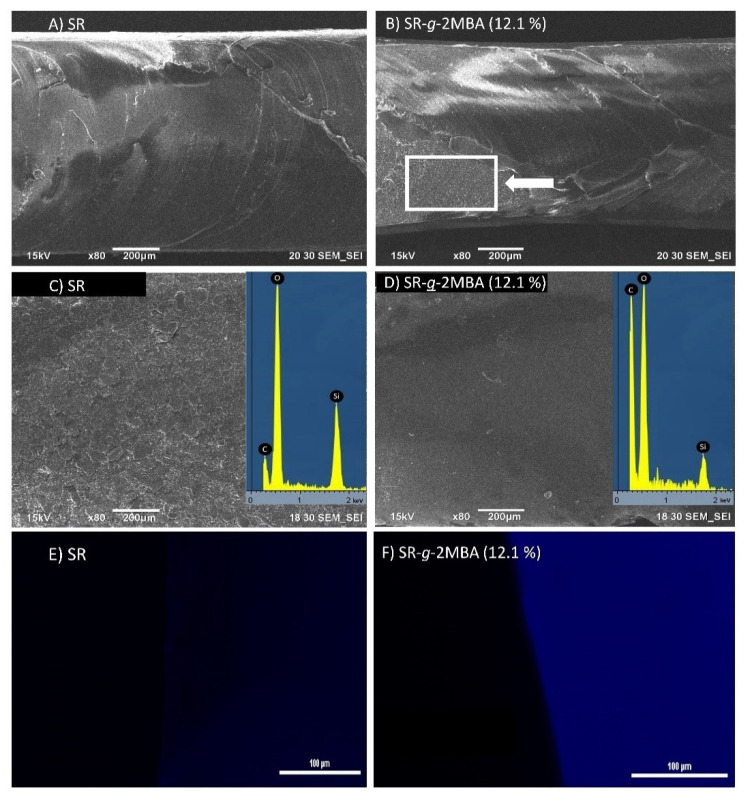
Scanning electron microscope (SEM) images of the cross-sections of (**A**) SR and (**B**) SR-*g*-2MBA 12.1%, the surfaces of (**C**) SR and (**D**) SR-*g*-2MBA 12.1%, and the surface fluorescence emitted by (**E**) SR and (**F**) SR-*g*-2MBA 12.1%. The increase in the intensity of the light emission (blue light) of functionalized film was observed.

We used scanning and fluorescence electron microscopy to corroborate whether the poly(2MBA) graft was made in bulk or only present on the surface of the SR. Cross-sections of pristine films were observed using SEM and showed homogeneity at the site (Figure 3A). However, in modified films, it was possible to observe sites or areas with differences in the morphology on the cross-section (Figure 3B, rectangle), corresponding to poly(2MBA) grafts in the bulk. There was a distinctive difference between the surface of the films with and without grafting (Figure 3C,D), which confirmed that poly(2MBA) was also grafted over the surface of SR. The similarities with bulk modifications were observed in grafts previously made of methacrylic acid on silicone [29]. 

Taking advantage of the light-emission properties of salicylate compounds previously reported [36], we performed fluorescence microscopy and observed that SR-*g*-2MBA film presents fluorescence (blue light), which is indicative of the presence of the salicylic acid derivative and which is not presented by unmodified film (Figure 3E,F). Another complementary test was the EDX analysis, which confirmed the presence of carbon, silicone, and oxygen on the surface of the SR films (Figure 3C). EDX of SR-*g*-2MBA demonstrated an increase in the proportion of carbon for the poly(2MBA) graft (Figure 3D).

#### 3.2.4. Salicylic Acid Release 

**Figure 4:**
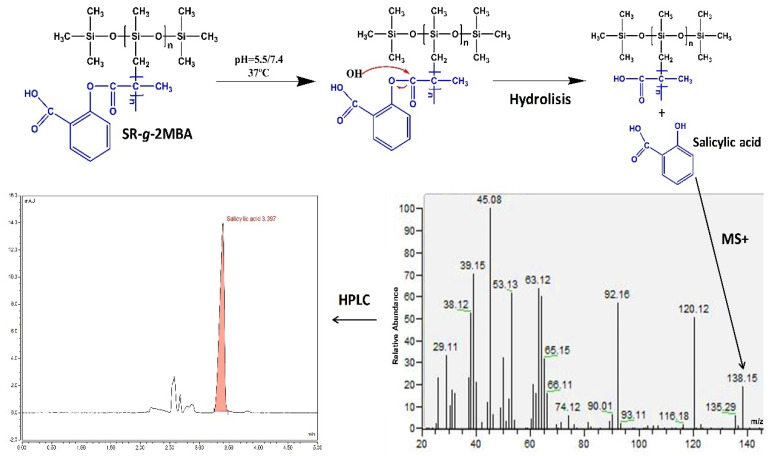
Schematic representation of SR-*g*-2MBA hydrolysis and salicylic acid release, molecular ion detection, and high-performance liquid chromatography (HPLC) quantification.

**Figure 5:**
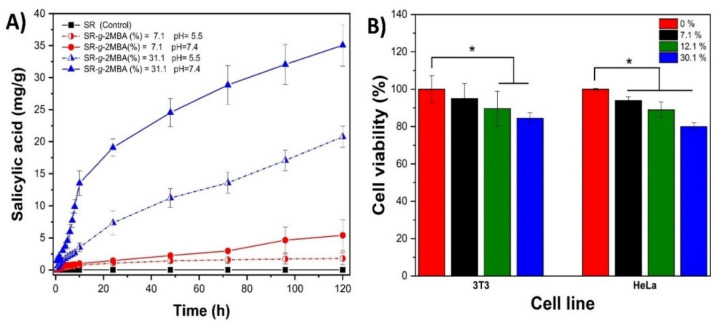
Release profile of salicylic acid from (**A**) SR-*g*-2MBA and viability of HeLa and BALB/3T3 cells after 24 h (**B**). The graphs represent the mean and standard deviation from three independent experiments, while the asterisk indicates statistical significance (*p* < 0.05).

From the release samples, the salicylic acid peak was observed on the HPLC chromatogram (Figure 4) with a retention time of 3.5 min, which is comparable to the retention time of the standard. The release media were taken, diluted with methanol, and subsequently injected into the GC-MS equipment. We observed the salicylic acid molecular ion, with its corresponding mass of 138.12 g/mol (Figure 4). The hydrolysis-release rate of salicylic acid from SR-*g*-2MBA was studied, and it was confirmed that the release was pH-dependent at buffer pH levels 5.5 and 7.4. A graft film at 7.1% demonstrated the lowest release rate of salicylic acid, which, at pH 5.5, reached a release of 1.77 mg/g at 120 h, with an increase in the release of up to 5.4 mg/g at pH 7.4 (Figure 5). This release difference was due to a higher susceptibility to hydrolysis at alkaline pH, caused by the higher solubility of salicylic acid at higher pH values [37,38].

Regarding high-graft-level films (31.1%), a greater drug quantification was observed at pH = 5.5 (20.8 mg/g at 120 h) and pH = 7.4 (35.1 mg/g at 120 h). The modified films showed two hydrolysis-release phases. The first was a rapid phase, observed in the first 10 h of quantification and apparently due to hydrolysis of the grafting surface. The second phase showed a slower rate, corresponding to the grafting within the bulk. 

The SR-*g*-2MBA released films showed zero- and first-order kinetics. The kinetic at a low graft level (7.1%) at pH 5.5 was adjusted to a zero-order model, while at pH 7.4 and 31.1% grafting (both pH values), it was adjusted to the first order. The kinetic adjustments and release constants can be seen in Table 1.

#### 3.2.5. Cytocompatibility 

The cytocompatibility of SR-*g*-2MBA films was evaluated through direct contact with HeLa (human) and BALB/3T3 (mouse) cell lines in small volumes of growing medium. Good cell viability (>90%) was demonstrated in low-graft films (7.1%) after 24 h of incubation time for both cell lines (Figure 5). In high-graft films (12.1% and 31.1%), we observed a significant (*p* < 0.05) reduction in the cell viability in both cell lines due to the high salicylic acid concentration released into the cell medium (47.5 mg/mL), as previously reported by Dasgupta et al. [26,38,39]. These results support future animal studies where factors such as physiological fluids, pH, blood perfusion rate, and volume must be taken into consideration for cytocompatibility [40]. 

## 4. Conclusions

The incorporation of the polymeric prodrug was achieved on SR by gamma irradiation, where monomer concentration and irradiation dose were key factors in the grafting percentage. Poly(2MBA) was grafted onto the surface and into the mass of the silicone. Salicylic acid was released by hydrolysis in a sustained and pH-dependent fashion, with low-graft-level silicone showing the best cytocompatibility. Gamma radiation is a powerful functionalization process for medical purposes, even though large-scale production would be a challenge. The results are encouraging for further testing of the low-graft-level materials for prophylactic use in handling inflammatory processes and bacterial colonization in cosmetic surgeries. The incorporation of this polymeric prodrug in medical devices or implants for breast reconstructions and augmentations could decrease the mean pain rating (currently 5.9 on a scale of 10), use of analgesics (mean 5.4 days), days off work (mean 6.6), and the 25-day period before returning to normal [41]. Implanting SR modified with the polymer prodrug poly(2MBA) could diminish recovery time, complication rates, and chronic infections. 

## Figures and Tables

**Table 1:** Release kinetics modeling.

Graft (%)	Release Medium pH	Kinetic Model	Rate Constants	R^2^
7.1	5.5	Zero order	0.0419 mg/g h	0.9853
31.1	5.5	First order	0.0166 h^−1^	0.9885
7.1	7.4	First order	0.0513 h^−1^	0.9289
31.1	7.4	First order	0.0246 h^−1^	0.9986
